# Genetic melting pot and importance of long-distance dispersal indicated in the *Gladiolus imbricatus *L. populations in the Polish Carpathians

**DOI:** 10.1038/s41598-021-96135-8

**Published:** 2021-08-17

**Authors:** Agnieszka Sutkowska, Józef Mitka, Tomasz Warzecha, Jakub Bunk, Julia Rutkowska, Roman Bathelt

**Affiliations:** 1 Department of Plant Breeding, Physiology and Seed Science, University of Agriculture in Krakow, ul. Podłużna 3, 30-239 Kraków, Poland; 2 Institute of Botany, Jagiellonian University, ul. Gronostajowa 3, 30-387 Kraków, Poland

**Keywords:** Ecology, Plant sciences, Ecology

## Abstract

The genetic diversity in 11 populations of *Gladiolus imbricatus* in five mountain ranges, including the Tatra, Pieniny, Gorce, Beskid Niski (Western Carpathians) and Bieszczady Mts (Eastern Carpathians), was studied with inter-simple sequence repeat (ISSR) markers. The species is a perennial plant occurring in open and semi-open sites of anthropogenic origin (meadows and forest margins). We checked a hypothesis on the microrefugial character of the plant populations in the Pieniny Mts, a small calcareous Carpathian range of complicated relief that has never been glaciated. Plant populations in the Tatra and Pieniny Mts had the highest genetic diversity indices, pointing to their long-term persistence. The refugial vs. the non-refugial mountain ranges accounted for a relatively high value of total genetic variation [analysis of molecular variance (AMOVA), 14.12%, p = 0.003]. One of the Pieniny populations was of hybridogenous origin and shared genetic stock with the Tatra population, indicating there is a local genetic melting pot. A weak genetic structuring of populations among particular regions was found (AMOVA, 4.5%, p > 0.05). This could be an effect of the frequent short-distance and sporadic long-distance gene flow. The dispersal of diaspores between the remote populations in the Western Carpathians and Eastern Carpathians could be affected by the historical transportation of flocks of sheep from the Tatra to Bieszczady Mts.

## Introduction

The Pieniny Mts (Polish Western Carpathians) are a relatively low range (Mt Wysoka 1050 m above sea level) spread on an area of ca. 100 km^2^-array that is part of the calcareous Pieniny Klippen Belt. The landscape is dominated by resistance to weathering rocks of Jurassic and Lower-Cretaceous origins, which contribute to the extraordinary richness of the site and microclimate conditions. It is located at a distance of only 20 km from the floristic center of the Tatra Mts. The uniqueness of the flora of the Pieniny Mts underlines the presence of endemics: Neogene *Taraxacum pieninicum* Pawł. and Pleistocene *Erysimum pieninicum* (Zapał.) Pawł. and glacial relicts *Dendranthema zawadzkii* (Herbich) Tzvelev and *Juniperus sabina* L. It has a rich flora with an estimated ca. 1,100 vascular plant species that constitute nearly 50% of the total Polish plant biodiversity^1^. Among the Western Carpathian ranges, the existence of an Eemian forest refugium is only postulated in Pieniny^2^.

It is known from historical records that the Carpathian ranges were colonized by the Wallachian tribes from the Balkans northward, who were accompanied by sheep breeds that can be dated back to the thirteenth and fourteenth centuries^3–6^. Wallachian settlers inhabited higher areas of the ranges with poorer soils that were unsuitable for cultivation, but enabled the continuation of the livestock and shepherding economy. In this way, Wallachian settlers created a permanent element of the Carpathian landscape. The sites where sheep and cattle were kept in pens and sites around shepherds’ huts are now dominated by anthropogenic, nitrophilous plant communities^7,8^.Traditional shepherding in the montane forest zone also led to the origin of unique meadow community *Gladiolo-Agrostietum capillaris assoc.*, growing on the shallow rendzinas of sites of previous lower montane beech forest^9^. This is an endemic plant association for the Western Carpathians. Extremely floristically rich overall, with 30 to 40 species per square meter and up to 70 species per 100 m^2^^10^, with the ecological center in the Pieniny Mts. Now, the plant association of the Pieniny Mts is being monitored within a Natura 2000 network of protected areas^11^. To protect it in the Pieniny National Park, cultural (traditional) sheep grazing is performed to increase species richness and maintain the region’s specific character^12^. The mountain meadows had their best ecological prospects in times of extensive agricultural-pastoral management. However, now that many of the areas that were previously used for economic purposes have been abandoned, the meadows have become rare and vulnerable.

One of the character species of the plant association is *G. imbricatus.* Before human activity opened the mountain landscape, it was probably characterized by ecotones between forest and rock sites, forest gaps (e.g., after fires), glades, rocky hillsides, balks, stony riversides, and alluvial forests^10^. Currently, *G. imbricatus* is disappearing in a wide array of plant communities across Central Europe^13^, including in the Pieniny Mts, where meadows with the character species have diminished from 300 ha in the 1960s to 35 ha in 2000^14^. Long-term regular extensive management is essential for the persistence of these populations^15^.

The main aim of the present study was to evaluate the molecular diversity of populations of *G. imbricatus* in the Western Carpathians. Especially, we attempt to (i) identify the region(s) with high genetic diversity of the species represented by their presumable refugial populations, (ii) identify the relationships between the Pieniny Mts and the neighboring Tatra and Gorce Mts (ca. 20 km), as well as the remote Beskid Niski and Bieszczady Mts (ca. 120–250 km), and (iii) discuss the phylogeography of *G. imbricatus* when compared with other species with a similar ecological profile.

The names of species follow Mirek et al.^16^ and the ipni.org database.

## Materials and methods

### Study species

The genus *Gladiolus* L. belongs to the Iridaceae family and has two distinct areas of geographic distribution. The main center is in Eastern and Southern Africa (Capensis), where more than 100 species have been described. The second area of distribution is in the southern part of Eurasia, especially in the Middle East and the Mediterranean region. In this area, the number of species is much smaller. Hamilton^17^ listed six species as indigenous to Europe. European species of *Gladiolus* are polyploids with a chromosome number from 60 in tetraploids to 180 in dodecaploids, but aneuploids there are known to exist. The evolution of the European *Gladiolus* species has been affected by hybridization and polyploidy. *Gladiolus imbricatus* covers Central and Eastern Europe, the Mediterranean, Caucasia, and West Siberia^13,17^.

*G. imbricatus* is an anemochore, perennial species, and a bulbo-tuber geophyte. It blossoms in July, pollinated by Lepidoptera. It occurs throughout Poland, but it is a rare species, more often inhabiting mountainous areas. Available historical data suggest that it was common in southern Poland in the nineteenth century^18–21^. It occurred in thermophilous oak forests of Potentillo albae-Quercetum, in moist lowland meadows of the Molinion alliance^22,23^, and as a weed in oat and barley fields^10,24^, probably as a remnant of previous meadow communities^18^. It was also a co-dominant species in many types of upland pastures and meadows^10,15,18^. This state has persisted until modern times; there has only been a drastic decline in the number of its sites in the last few decades. This process is particularly visible in the central and northern parts of the country, where the appearance and disappearance of new populations is observed^25,26^. In southern Poland, population declines and fragmentation are also recorded. As a result, *G. imbricatus* is now endangered in Poland^27^. Moreover, it is gradually disappearing in the whole of Europe^13,23,28^. In Poland, the plant species is under law protection.

### Sample collection

**Figure 1:**
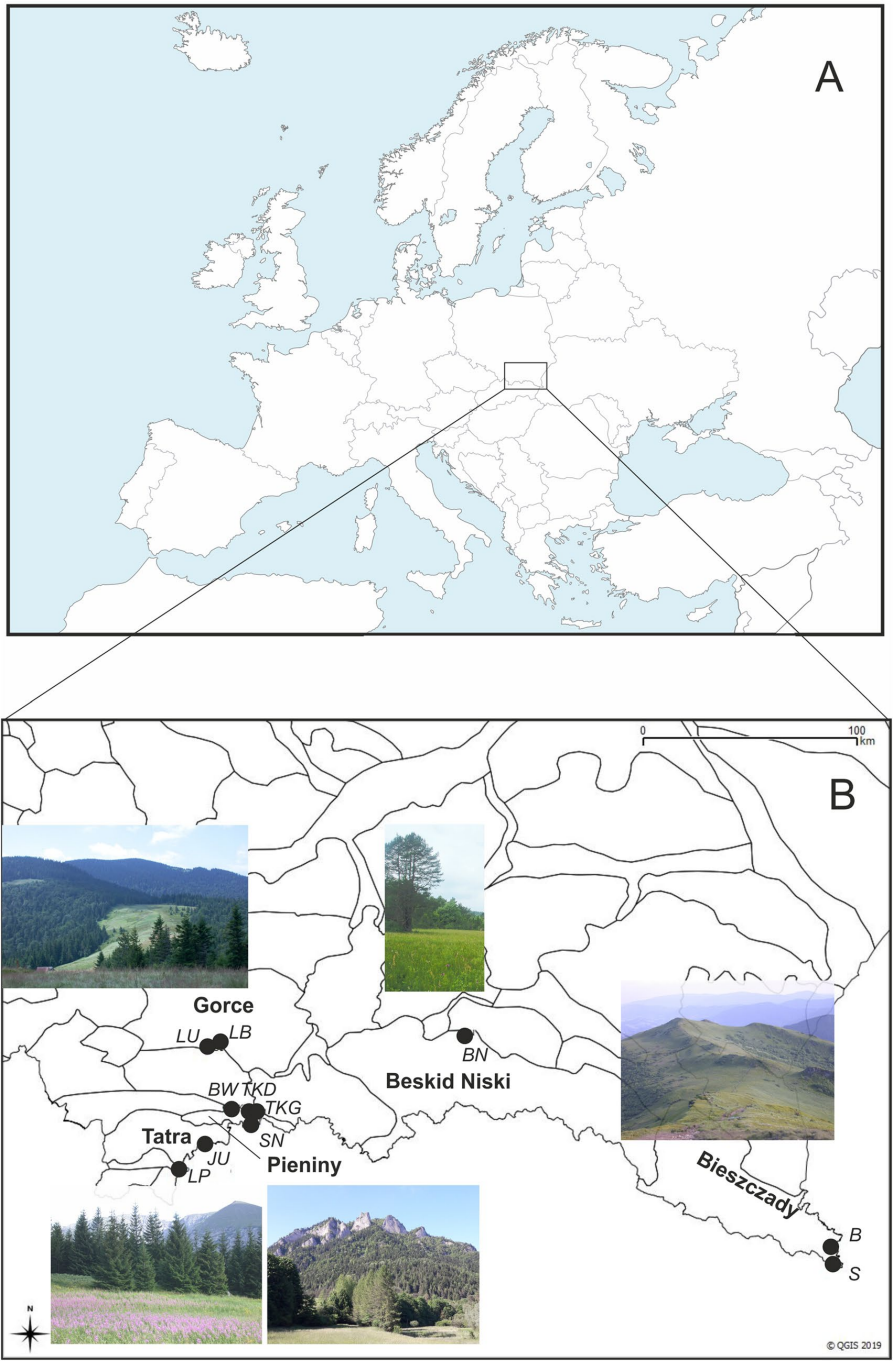
Location of the studied area on a map of Europe (**A**). Distribution of sampling locations of *Gladiolus imbricatus* in Polish Carpathians (**B**). For abbreviations of localities see Table 1. Inserts: Mountain landscapes with semi-natural meadow communities: Gorce, Tatra, Pieniny, Beskid Niski, and Bieszczady Mts. Maps generated with QGIS Version 3.20 (https://www.qgis.org/). Figure, photos and map by J.M.

Plant material (fragments of ca. 4 cm^2^) to DNA isolation was collected in nine populations in the Western Carpathians and in two populations in Eastern Carpathians (Bieszczady Mts, Table 1). Populations *TKG*, *TKD*, and *BW* represent moderately dry variant of Pieniny’s meadow *Gladiolo-Agrostietum capillaris*; *JU—*nitrophilous dry meadow with the domination of *Linaria vulgaris*; *LU, LB* (Gorce), *LP* (Tatra) *S, B* (Bieszczady), *BN* (Beskid Niski Mts) represent wet meadows of Molinion caeruleae alliance (Fig. 1, Table 1).

**Table 1:** Locations of *Gladiolus imbricatus* taken for the study in the Polish Carpathians.

Locality	Abbreviation	Sample size	Plant community	GPS position	Altitude m.s.l
**Pieniny Mts**					
Trzy Korony 1	TKG	9	*Gladiolo-Agrostietum *(dry meadow)	N 49 24 E 20 24	450
Trzy Korony 2	TKD	9	*Gladiolo-Agrostietum *(dry meadow)	N 49 24 E 20 23	570
Sromowce Niżne	SN	2	Fallows	N 49 23 E 20 23	489
Jaworki	BW	9	*Gladiolo-Agrostietum *(dry meadow)	N 49 24 E 20 34	621
**Gorce Mts**					
Lubomierz 1	LU	7	*Cirsietum rivularis* (wet meadow)	N 49 36 E 20 14	663
Lubomierz 2	LB	4	*Cirsietum rivularis* (wet meadow)	N 49 36 E 20 14	651
**Tatra Mts**					
Łysa Polana	LP	7	*Cirsietum rivularis* (wet meadow)	N 49 16 E 20 07	984
Jurgów	JU	14	*Gladiolo-Agrostietum/Hieracio-Nardetum* (dry meadow)	N 49 18 E 20 09	847
**Bieszczady Mts**					
Sianki	S	5	*Cirsietum rivularis* (wet meadow)	N 49 01 E 22 51	822
Beniowa	B	3	*Cirsietum rivularis* (wet meadow)	N 49 03 E 22 51	730
**Beskid Niski Mts**					
Bednarka	BN	8	Molinion (wet meadow)	N 49 38 E 21 18	381

The sampling of *G. imbricatus* under national law according to Biodiversity Act, 2004 (Dz. U. 2014, no. 1409) and according to Convention on the Conservation of European Wildlife and Natural Habitats (Bern Convention) (1979). The sampling in the Bieszczady National Park was permitted by document no. 60/17. According to Polish law, it does not require the permission of the relevant institutions when we collect small part of leaves outside the area of National Park. The formal identification and collection of the plant material used in our study by authors: A. Sutkowska (A.S.) and J. Mitka (J.M.). Voucher specimen from the site in Beniowa (Bieszczady), stored in the herbarium of the Bieszczady National Park at ID 1503 (the formal identification by W. Paul). DNA samples of all examined specimens are available from the author (A.S.) upon request.

### DNA isolation and ISSR analysis

DNA was isolated from fully developed fragments of leaves (4 cm^2^) without damage symptoms caused by insects and mold. DNA was extracted with Genomic Mini AX Plant (A&A Biotechnology) according to the manufacturer's instructions. The sequences of primers were taken from Stepansky et. al.^29^ and are shown in Table 2. PCR reactions were conducted with a 2720 Thermal Cycler (Applied Biosystems). The composition of the reaction mixture and the PCR reaction conditions according to Gupta et al.^30^ and optimized by Sutkowska^31^. Amplification was carried out with 25 µl reaction mixture: 2.5 µl tenfold concentrated reaction buffer supplied by the Taq DNA polymerase manufacturer (Fermentas), 1.5 mM MgCl_2_, 0.19 mM of each dNTPs (Fermentas), 27 pmol primer, 200 ng template DNA and 1.4 units of Taq polymerase. The annealing temperature for primers ISSR7 was 44 °C, and for ISSR1, ISSR3, ISSR6 was 47 °C. Optimal conditions for the reaction were as follows: initial denaturation: 94 °C—5 min, 42 amplification cycles: denaturation 94 °C—59 s, annealing 44 °C (47 °C)—59 s, polymerization 72 °C—59 s, final polymerization 72 °C—7 min. A negative control reaction without DNA template was included in each amplification. To confirm the results, 50% of the samples were amplified twice.

**Table 2:** The primers used in PCR, primer sequence, total number of reactions products generated by each primer and range fo number of PCR product per specimens.

Primer	Primer sequence	Number of PCR products	Number of PCR products per specimen
ISSR 1	(TC)_8_C	49	4–11
ISSR3	(GGGTG)_3_	40	4–9
ISSR6	(AC)_8_G	41	5–13
ISSR7	(AC)_8_T	35	3–12

Products were subjected to electrophoresis on 1.5% agarose gel stained with ethidium bromide (50 µl/100 ml) at 100 V about 1.5 h. PCR products (bands patterns) were observed and archived with Imagemaster VDS (Pharmacia—Amersham).

For analysis of the length of PCR products, GelScan ver. 1.45 (Kucharczyk TE) software was used. ISSR reproducibility tests included within-plate (n = 12) and between-plate (n = 9) replicates independently analyzed from the DNA extracts^32^.

### Data analysis

All the analyses performed were based on the following assumptions: ISSR markers behaved as dominant markers; co-migrating fragments were considered homologous loci; and populations were at Hardy–Weinberg equilibrium, in which case allele frequencies were estimated from Bayesian method with the non-uniform prior distribution of allele frequencies, to calculate expected heterozygosity *Hj*. Statistics of genetic diversity and population genetic structure were computed after estimating allele frequencies, including the percentage of polymorphic bands (*PLP*) at 95% criterion. The calculations were performed with AFLP-SURV version 1.0^33^.

Shannon’s diversity index (*I*) was calculated to provide a relative estimate of the degree of genetic variation within each population, based on the formula *I* = -Σ Pi log2 Pi, where Pi was the frequency of each ISSR band. The number of alleles (bands) with nonzero frequency (allele number *na*) and the reciprocal of homozygosity (effective allele number *ae*) were calculated using POPGENE version 1.32^34^. As an additional diversity marker, the rarity index *DW*, corresponding to “frequency-down-weighted marker values” per individual^35^ was computed using AFLPdat^36^. High *DW* values are expected in long-term isolated populations^37^. Analysis of molecular variance (AMOVA)^38^ was carried out using the program Arlequin version 3.11^39,40^.

Matrix of Nei's genetic distance^40^ was calculated for each pair of 11 populations with AFLP-SURV^33^. The matrix was then used to construct an NJ tree^41^ with reticulations with the T-REX ver. 4.1 software^42^, and the stopping rule for reticulations based on Criterion Q1^43^. The bootstraps were calculated in CONSENSE option implemented in PHYLIP version 3.6^44^ based on 1000 random runs.

The matrix of 77 samples × 167 ISSR products coded as binary (0–1) data was used to calculate Nei and Li (Dice) distances^45^. They were used for a non-metric multidimensional scaling analysis (NMDS)^46^ performed with the NTSYSpc ver. 2.11 multivariate analysis package^47^. NMDS displays a monotone relationship to the distances implied by the original data matrix and achieves a much better fit in fewer dimensions than is possible with other ordination methods.

The genetic division among individuals in populations of both species was estimated using STRUCTURE, version 2.3.3^48^, applying a Bayesian model-based clustering algorithm for the use of dominant markers^49^. The numbers of K = 1–5 groups were tested in ten replications per K. A burn-in period 100 000 was applied, followed by a procedure using 500 000 Markov chain Monte Carlo (MCMC) repetitions. The estimation of the optimal number of groups was based on the likelihood of partitions, estimates of posterior probability provided in STRUCTURE output, examined as a function of increasing K^48^ and ΔK values, estimating the change in the likelihood function with respect to K and estimated as an indicator of the most reliable clustering structure^50^. The similarity between runs was estimated using the symmetric similarity coefficient^51^ with the R-script Structure-sum-2011^36^. K = 2 represented an optimal clustering structure.

## Results

In the ISSR analysis of 77 individuals with 4 primer combinations, 165 unambiguous fragments (loci) were selected ranging between 130 and 990 bp. The number of PCR products per individual varied in particular primers within 3–13 (Table 2). The within-population genetic variation was calculated using different indices (Table 3).

**Table 3:** Population genetic diversity of *Gladiolus imbricatus* in Western Carpathians and Eastern Carpathians (Bieszczady) and distribution of private and inter-regional specific bands based on 167 ISSR products.

Population	*n*	*na*	*ne*	*PLP*	*Hj*	*SE(Hj)*	*I*	*DW*	No. of specific bands (loci)**
**Bieszczady Mts**									
*B*	3	1.131	1.078	22.6	0.087	0.013	0.071	1.745	2^a^
*S*	5	1.327	1.170	36.3	0.160	0.014	0.160	2.380	2^b^
Total	8	1.381	1.182	39.9	0.150	0.014	0.172	2.148	–
**Beskid Niski Mts**									
*BN*	8	1.381	1.158	42.3	0.153	0.013	0.164	1.910	2^c^
**Gorce Mts**									
*LB*	4	1.256	1.150	29.2	0.139	0.014	0.134	1.908	–
*LU*	7	1.298	1.139	36.9	0.119	0.013	0.134	2.337	2^c^
Total	11	1.458	1.192	47.0	0.153	0.014	0.188	2.187	–
**Tatra Mts**									
*LP*	7	1.292	1.133	36.9	0.121	0.013	0.130	2.104	2^c^
*JU*	14	1.440	1.154	36.9	0.138	0.012	0.164	2.785*	–
Total	21	1.679	1.184	55.4	0.194	0.011	0.216	2.578*	–
**Pieniny Mts**									
*SN*	2	1.077	1.054	22.6	0.077	0.012	0.049	1.906	–
*TKD*	9	1.327	1.136	38.1	0.120	0.013	0.137	1.469	2^b^, 2^d^
*TKG*	9	1.387	1.164	43.5	0.151	0.013	0.167	1.917	–
*BW*	9	1.417	1.192	44.0	0.169	0.013	0.187	2.672*	2^a^
Total	29	1.679	1.201	50.6	0.189	0.011	0.227	2.048	–

*DW* rarity index corresponding to “frequency-down-weighted marker values” per population, *Hj* Nei’s gene diversity, *SE(Hj)* standard error of *Hj*, *I* Shannon’s information index, *n* sampling size, *na* observed no. of alleles, *ne* effective no. of alleles, *PLP* percentage of polymorphic loci. See also Table 1.

*Significantly (p < 0.05) higher value based on 999 permutations.

**The same letter denotes common occurrence of the same bands in various populations.

All genetic variation values were consistently highest in populations Tatra *JU* and Pieniny *BW*. The mean allele number *na* and the effective number of alleles *ne* ranged between 1.077–1.440, and 1.078–1.192, respectively. The mean percentage of polymorphic loci (*PLP*) ranged between 22.6 and 44.0%, and Shannon’s *I* index was between 0.049 and 0.187. Higher values for genetic variation were recorded in the Tatra Mts, then in the Pieniny Mts. Lower values were found in the Gorce and Bieszczady Mts. The survey of the rarity index *DW* revealed a similar pattern of variability across regions. Its highest values were found in Tatra *JU* and Pieniny *BW*, and the lowest in Beskid Niski *BN* and Gorce *LB*.

**Figure 2:**
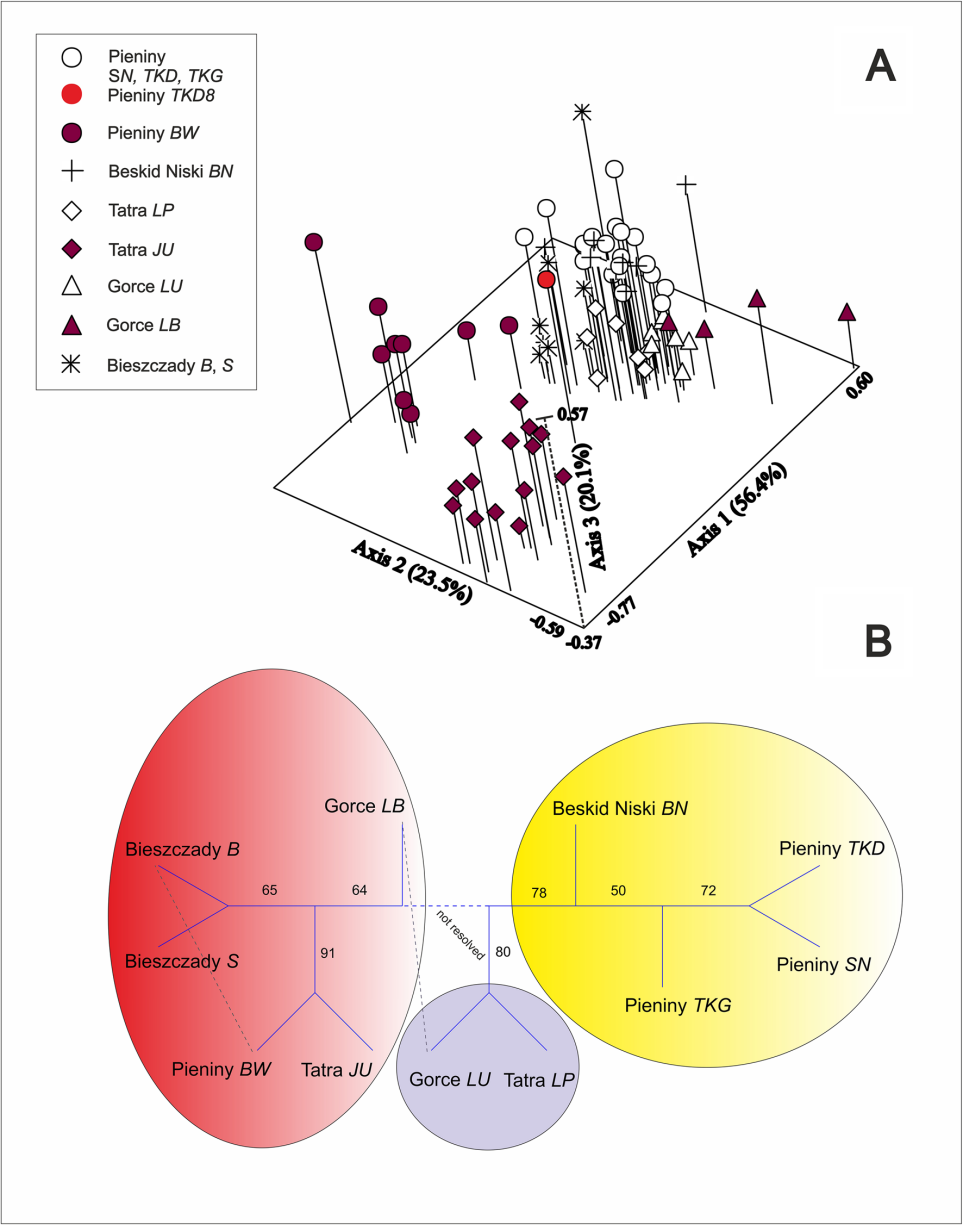
Results of numerical analyses of 11 populations and 77 individuals of *Gladiolus imbricatus* in Polish Carpathians based on 168 ISSR bands. **(A)** Non-metric Dimensional Scaling (NMDS, Dice distance, NTSYSpc^47^); **(B)** neighbour joining (NJ) classification with reticulations (T-REX^42^). The unrooted tree is based on Nei’s distance. Figure by J.M.

The NMDS ordination based on ISSR variability displayed three main genetic groups of *G. imbricatus*: (1) Pieniny *BW*, (2) Tatra *JU*, and (3) the remaining individuals (Fig. 2A).

The first group from the Pieniny *BW* formed individuals of the hybridogenous origin, according to the results of the Bayesian STRUCTURE analysis. Also, a hybridogenous is an individual Pieniny *TKD*8 (STRUCTURE—result not shown, Fig. 2A). All hybrid individuals have genetic admixture from the population Tatra *JU*. The populations Pieniny *BW* and Tatra *JU* formed a highly (91%) supported sister group (NJ, Fig. 2B). The second group of Tatra *JU* is genetically heterogenous: four individuals show the links with the population Pieniny *TKD* and, to a lesser extent, with Bieszczady *S*. The third group forms the remaining populations. Among them, the fairly uniform Tatra-Gorce *LP/LU* group is visible, with high support of 80% (NJ). The second population Gorce *LB* is presumably of the hybridogenous origin, with the admixture of Tatra *JU* (STRUCTURE—result not shown, Fig. 2A).

**Figure 3:**
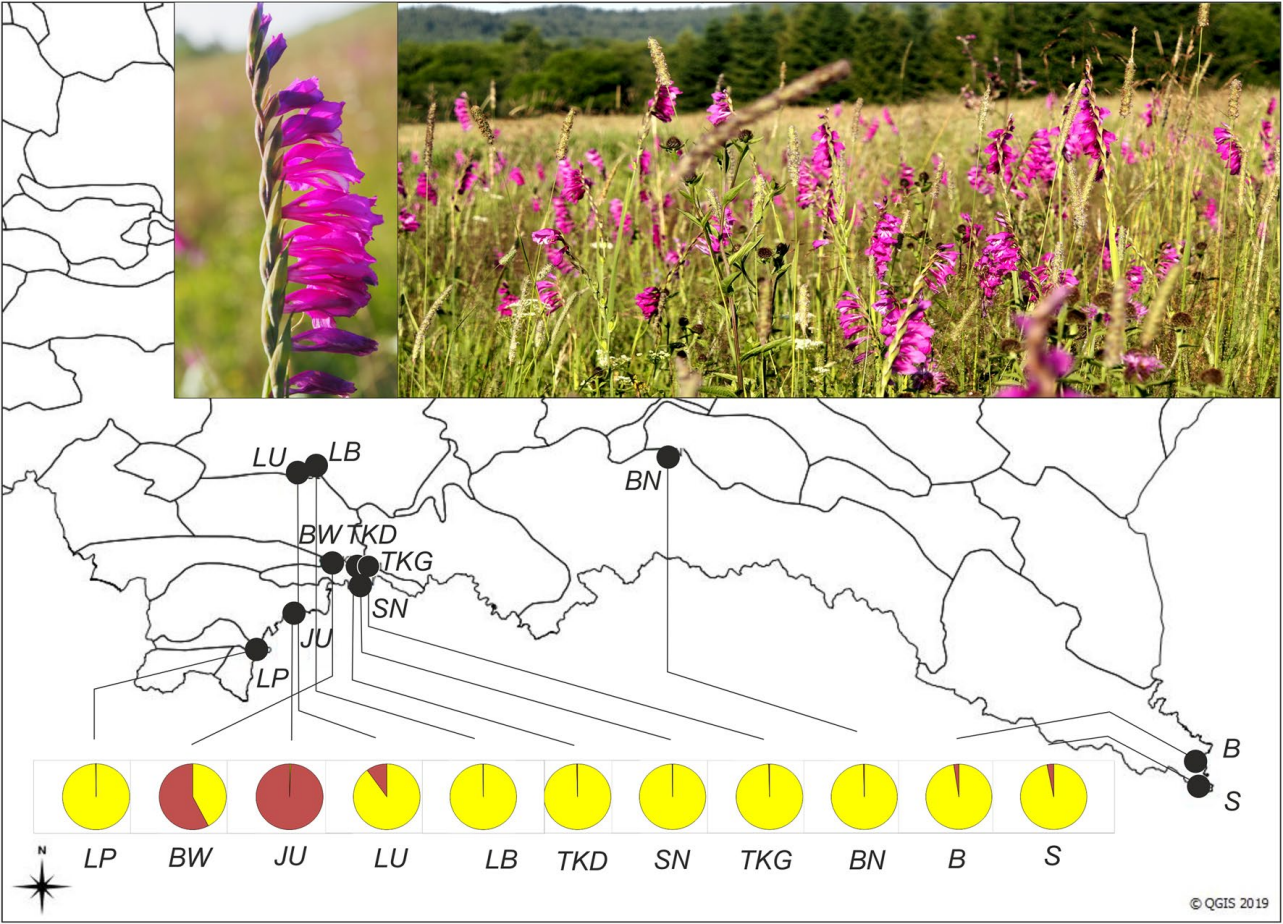
Results of Bayesian clustering (STRUCTURE^48^) of ISSR genetic diversity in populations of *Gladiolus imbricatus* in Polish Carpathians. Pies charts show the estimated probability of occurrence of a genetic group depicted by its color. Note populations in Bieszczady B and S (E Carpathians) which display genetic imprints from population Tatra JU (W Carpathians). Map generated with QGIS Version 3.20 (https://www.qgis.org/). Insert: *G. imbricatus* in Bieszczady, Beniowa. Figure, photos. and map by J.M.

The results of the NJ classification with reticulations show the existence of the three clusters (Fig. 2B). The first group, with a moderate support of 64%, consists of Pieniny *BW* and Tatra *JU* (highly supported with 91%), Bieszczady *B, S*, and Gorce *LB.* Here a reticulation between Pieniny *BW* and Bieszczady *B* was found. The second group forms an unresolved cluster with Gorce *LU* and Tatra *LP* (80% support). The third group consists of the remaining Pieniny populations *TKD, SN, TKG* (50% support) and the population from Beskid Niski *BN* (78% support, Fig. 3).

A reticulation between Gorce *LB* and Gorce *LU* was noted.

Bayesian analysis (STRUCTURE, K = 2) show the distribution of genetic diversity among populations (Fig. 3). The genetically most specific was population Tatra *JU*. Population Pieniny *BW*, and in a lesser extent Gorce *LU* were hybridogenous. Two remote Bieszczady’s populations *B* and *S* had a small admixture of the genetic diversity from Tatra *JU*/Pieniny *BW/Gorce LU* (Fig. 3).

The above genetic links between geographically remote populations are supported by the occurrence of specific bands (alleles), occurred exclusively in two or three geographical regions. The populations from Bieszczady Mts *B* shared two unique bands with Pieniny *BW* and two unique bands (Bieszczady *S*) with Pieniny *TKD*. The NJ group Gorce *LU/*Tatra *LP* shared two unique bands with Beskid Niski *BN*. The only population with two private bands was Pieniny *TKD* (Table 3).

Non-hierarchical AMOVA showed a significant but moderate level of genetic divergence among 11 populations of *G. imbricatus*: 37.61% (p < 0.001). The analysis of the genetic structure of the populations in five geographical regions showed small, statistically insignificant, population differentiation in the particular mountain ranges (4.50%, p > 0.05). The test a hypothesis on the genetic divergence of the populations in two presumably refugial ranges: Pieniny and Tatra Mts gave the statistically significant result: 14.12% (p = 0.003) (Table 4).

**Table 4:** Analysis of molecular variance (AMOVA) of *Gladiolus imbricatus* in the Polish Carpathians.

Level of variation	d.f	Sum of squares	Variance components	Percentage of variation	P value
**A. All populations**					
Among populations	10	595.936	7.004	37.61	< 0.001
Within populations	66	766.973	11.620	62.39	
Total	76	1362.909	18.625		
**B. Among geographical regions**					
Among regions	4	282.696	0.845	4.50	> 0.05
Among populations within regions	6	313.240	6.299	33.57	
Within populations	66	766.973	11.621	61.93	
Total	76	1362.909	18.765		
**C. Refugial vs. no refugial regions**					
Among regions	2	237.352	2.743	14.12	0.003
Among populations within regions	8	358.684	5.067	26.08	
Within populations	66	766.973	11.621	59.81	
Total	76	1362.909	19.431		

A—with no geographical structuring; B—with 5 geographical regions; C—with 2 refugial (Pieniny and Tatra Mts) vs. 3 no refugial regions (Gorce, Beskid Niski, Bieszczady Mts).

## Discussion

Open-site natural vegetation forms peculiar plant communities in high-mountain zones (alpine vegetation), northern latitudes (arctic tundra belt), and continental climates (steppe formations). Historically, it also occurred in the Carpathian forelands and adjacent lowlands in the Late Vistulian (the Oldest Dryas), forming the tundra and cold steppe formations, according to many pollen spectra. No pollen of Iridaceae was found at that time in Poland^52^. Our results show that *G. imbricatus* could have persisted locally in the Last Glacial Maximum in the Tatra Mts and Pieniny Mts (Western Carpathians). The latter calcareous mountain range could have formed a local refugium and genetic melting pot for many plant species.

Our study revealed the high genetic variability of *G. imbricatus* in the Western Carpathians and the flow of alleles between both neighboring and distant populations. A study on *G. imbricatus*^53^ also showed the low percentage of polymorphic bands *PLP* in populations of the species located about 100 km more to the north in Poland (25–26%). Our study showed the value of *PLP* within 40–55%. Nei’s genetic diversity index *Hj* was also higher in the Carpathians (0.150–0.194 vs. 0.084–0.100). The among-population component of diversity [analysis of molecular variance (AMOVA)] amounted to 36.6% in the Carpathians and 22.0% in populations located further north in Poland^53^. The similar genetic diversity indices *PLP* and *Hj* found at *G. imbricatus* in the vicinity of Minsk (Belarus)^54^, and amounted to 38%, and 0.130, respectively. The reason for such high genetic diversity is unknown; the population was perhaps related to an unrecognized “cryptic” refugium nearby.

Generally, the higher genetic diversity of Carpathian populations compared to those in lowland areas could be an effect of their refugial character (especially in the Pieniny and Tatra Mts). For AMOVA, these two geographical regions accounted for 14% (p = 0.003) of the total genetic variance of the Carpathian populations of *G. imbricatus*. The result showed the statistically significant genetic divergence between refugial and non-refugial areas, with the outcome being especially convincing when compared with the non-significant structuring of the populations in five geographic regions of the Polish Carpathians. Populations of the species localized more to the north in Poland seem genetically impoverished in comparison with those occurring in the mountains. Similar results concerning the lowland vs. mountain populations showed studies on forest grass *Bromus benekenii*^55^ and wet meadow *Primula farinosa*^56^, which could have originated from the glacial refugia, especially those located in the Carpathian refugium^57^.

A much higher among-population component of diversity (AMOVA) that amounted to 86% displayed a grass, *Melica transsilvanica*, studied in the Pieniny Mts and Małopolska Upland (southern Poland)^58^. The species has a different ecological profile than *G. imbricatus*; it grows on dry, exposed outcrops in pioneer rupicolous grasslands (*Seslerio-Festucion duriusculae* alliance in the Pieniny Mts) and in xerothermic grasslands of the *Cirsio-Brachypodion pinnati* alliance on the Małopolska Upland. The high level of among-population diversity was linked with a decreased within-population diversity index (*PLP* 3.7%) and nearly total genetic homogeneity. Taken together, the results of the studies on *M. transsylvanica* in the Pieniny Mts point to the high geographical isolation of the metapopulation in the region and the dominant self-pollinating system of the species is inducing the lack of gene flow^58^.

The relatively high within-population genetic diversity of the *G. imbricatus* populations in the Carpathians could be related to the presumably refugial character of some Western Carpathian populations. This has been proven by the occurrence of the private bands, a typical phenomenon in the relict areas^59^, and the high value of the *DW* index^37,60^. Accordingly, the high genetic diversity could be the effect of the long-time presence of large population numbers in the interglacial^15^, counteracting the effects of genetic drift or genetic bottleneck that affect smaller populations more heavily. An example is the extant Polish population of *Primula farinosa* in the Pieniny Mts which probably formed a continuous distribution in the interglacial between the Carpathian Mts and Polish lowlands^56^. Today, the relict plant population demonstrates a distinct genetic makeup and admixture of various genetic lineages, an example of the Pieniny genetic melting pot. Increased diversity was obtained through the redistribution of genetic information in a given place by dispersal from refugial areas^55,59,60^. Our study of *G. imbricatus* also provides clear phylogeographic evidence on the existence of a local genetic melting pot in the Pieniny Mts. The Bayesian inference shows the two genetic types of the species in the Polish Carpathians. They formed a hybridogenous population Pieniny *BW*, the sister group, with a donor population Tatra *JU*. In the other population, Pieniny *TKD*, one individual (*TKD8*) also shows the genetic admixture from Tatra *JU*. It is compelling evidence of the local short-distance migrations. Moreover, frequent short-distance dispersal could probably prevent the effects of genetic drift (genetic bottleneck) in the distracted and divided populations of southern Poland.

The remaining populations in the Pieniny Mts formed a distinct but marginally supported cluster (50%), showing the different histories of their origin. The same concerns apply to two populations in the Gorce Mts. Geographically adjacent, they have not originated from the same genetic stock; the first (Gorce *LU*) has close genetic links with Tatra *LP* (NJ), and the second (Gorce *LB*) with Tatra *JU* (STRUCTURE, NMDS). Interestingly, the most remote populations, Bieszczady *B* and *S*, were linked genetically to the Pieniny *BW* population, as well as with Gorce *LU* and Tatra *LP* (a distance of ca. 250 km). The reticulation in NJ and the sharing of the rare specific alleles in such remote populations as Bieszczady *B* and *S*, and Gorce/Tatra group *LU/LP*, together with Pieniny *BW, TKD, SN*, point to the occurrence of long-distance dispersal. The placement of the population from Beskid Niski *BN* in one group with Pieniny populations could characterize the stochastic process.

The genetic similarity of the distant populations (Bieszczady vs. Tatra Mts) may be a result of numerous overlapping factors, including multidirectional gene flow in the dispersal history, long-distance dispersal during postglacial recolonization, and survival in several detached refugia^61^. All of these factors led to diminishing the among-population diversity component. We found rare, shared bands in remote populations that can most likely be explained by long-distance migration. The relevant example is the close genetic links between the Pieniny/Tatry and Bieszczady Mts. The long-distance migration in *G. imbricatus*, which has limited dispersal ability^62^, could be explained by human impact. For example, the transport of numerous sheep flocks (up to 50,000 individuals) by rail from the Tatra Mts to the Bieszczady Mts in the 1960s was the result of overgrazing in the Tatra Mts area, and the administrative decision of the Tatra National Park authorities^64^. Also, many villages in the Beskid Niski Mts were abandoned after the Second World War and the flocks of sheep from the Tatra/Pieniny regions were then introduced there. In our opinion, this could be a crucial factor in determining the close genetic relations between populations in such remote regions.

The similarities among populations in close areas of the Pieniny, Tatra, and Gorce Mts, where sheep flocks were abundant, densely distributed, and interchanged often in historical times, could be explained by short-distance diaspore dispersal.

While studying the genetic diversity of *Aconitum s*pecies in the Carpathians and the Sudetes^63^, an introgression was found between species with non-overlapping geographical ranges, probably as a result of seed dispersal with pasturing within a distance of ca. 20 km (Mantel test). It seems to be an average of short-distance seed dispersal in the mountains, in the realm of the traditional agro-pastoral economy.

The lack of genetic differences between populations in different regions is a common phenomenon in phylogeographic studies. For example, in a study on the genetic diversity of a subalpine perennial *Cicerbita alpina* across Europe^65^, the authors found a weak phylogenetic structure of the tall-herb community species among Carpathians regions (Eastern Carpathians, Southern Carpathians, Western Carpathians) (AMOVA among-group component equaled 9.78%). Also, the level of genetic divergence of the species (30%) in the non-hierarchical analysis is lower than an average in the case of mountain species. The result was explained by the biological characteristics of the species: a tall plant (up to 2.5 m), producing a large number of small diaspores over long distances, can promote gene flow between geographically distant regions and result in low parameters of genetic isolation.

Summing up, glacial refugia of *G. imbricatus* could have existed in the Western Carpathians. An example is the population Tatra *JU*, the most genetically diversified population forming a distinct genetic group among the populations that have been studied. A hybridogenous population was found in the Pieniny Mts, which are generally believed to be a glacial refugium, proving the dispersal from the Tatra Mts and the existence of the local genetic melting pot. The other dispersal from the Tatra Mts refugium was also traced adjacent to the Gorce Mts. The unexpected genetic links found between the remote populations of *G. imbricatus*, namely the Pieniny/Tatra and Bieszczady populations, could be explained by long-distance dispersal linked with the transportation of sheep flocks between the Tatra and Bieszczady Mts in the second half of the previous century. The migrations of flocks were accompanied by the plants (diaspores), which facilitated the mixing of hitherto isolated populations. In effect, a lowering of the values of genetic divergence between distant populations of *G. imbricatus* was noted. In this way, the genetic links between plant populations in close or distant mountain ranges could be affected by the recent, stochastic processes leading to the decreasing values of genetic divergence indices. In conclusion, the role of many centuries of agro-pastoral human economic activity in the migration of the plant species seems, in some cases, crucial.

## Data Availability

Data on genetic variation in *Gladiolus imbricatus* are available from the authors upon request.
